# Trends in Concurrent Psychiatric Comorbidities in Alcohol-Associated Liver Disease: A Nationwide Study from 2015–2023

**DOI:** 10.1007/s10620-025-09494-x

**Published:** 2025-11-06

**Authors:** Shu-Yen Chan, Yee Hui Yeo, Hyunseok Kim, Molly Delk, Natchaya Polpichai, Pojsakorn Danpanichkul, Peng-Sheng Ting

**Affiliations:** 1 Department of Internal Medicine, Vanderbilt University Medical Center, Nashville, TN USA; 2 Karsh Division of Gastroenterology and Hepatology, Department of Medicine, Cedars-Sinai Medical Center, Los Angeles, CA USA; 3 Karsh Division of Gastroenterology and Hepatology, Cedars-Sinai Medical Center, Los Angeles, CA USA; 4 Division of Gastroenterology and Hepatology, Tulane University School of Medicine, New Orleans, LA USA; 5 Division of Gastroenterology & Hepatology, Medical College of Georgia, Augusta University, Augusta, GA USA; 6 Department of Internal Medicine, Texas Tech University Health Sciences Center, Lubbock, TX USA; 7 University Medical Center of New Orleans, New Orleans, LA USA; 8 Assistant Professor of Medicine, Director of Hepatology Research, Dept of Gastroenterology, Tulane University School of Medicine, 131 S. Robertson St, Suite 800, New Orleans, LA 70112 USA

**Keywords:** Alcohol-associated liver disease, Chronic liver disease, Psychiatric comorbidities, Cirrhosis, Anxiety disorder, Major depressive disorder (MDD), Post-traumatic stress disorder (PTSD)

## Abstract

**Background:**

Psychiatric comorbidities with alcohol use disorder increase the risk of alcohol abuse and relapse. However, updated epidemiologic trends of comorbid psychiatric disorders in patients with alcohol-associated liver disease (ALD) remain limited. We aim to investigate the trends of prevalence of psychiatric comorbidities among those with ALD.

**Methods:**

This population-level cohort study used TriNetX data from over 113 million U.S. patients to assess trends in nine psychiatric disorders from 2015 to 2023 among individuals with ALD, stratified by cirrhosis status and age. In addition, we also compared the trends between patients with ALD and non-ALD chronic liver disease (CLD). Joinpoint regression analyses evaluated temporal trends using annual percentage changes (APC) and average annual percentage changes (AAPC) to identify significant inflection points over the study period.

**Results:**

We observed significant increases in the prevalence of major depressive disorder (MDD), anxiety, and post-traumatic stress disorder, while schizophrenia and psychotic disorders declined in both ALD with and without cirrhosis groups. In individuals with ALD cirrhosis, schizophrenia and psychotic disorders declined at a greater rate than those without cirrhosis. Stratifying by age, we found older individuals with ALD have a rising trend in seven out of nine psychiatric comorbidities and had a greater increase compared to younger individuals, particularly in MDD, anxiety, and bipolar disorder.

**Conclusion:**

Patients with ALD without cirrhosis have the highest psychiatric comorbidity rates. However, trends of prevalence are rising significantly among those with ALD cirrhosis and older adults, highlighting the urgent need for targeted mental health interventions in this vulnerable population.

**Supplementary Information:**

The online version contains supplementary material available at 10.1007/s10620-025-09494-x.

## Background

Alcohol-associated liver disease (ALD) is one of the most common forms of chronic liver disease (CLD) and a leading indication for liver transplantation. Globally, ALD accounted for the loss of approximately 11 million life-years in 2019 [1, 2]. In the United States, the American College of Gastroenterology estimates ALD affects 3.5% of the general population and up to 55.1% of individuals with alcohol use disorder [3]. ALD also accounts for 48% of cirrhosis-related death [4] and 41.1% of liver transplant listings nationwide in 2023 [5]. Patients with ALD have a higher prevalence of psychiatric comorbidities, including depressive episodes, severe anxiety, insomnia, suicidality, and polysubstance use disorders [6–8]. The presence of psychiatric comorbidities among ALD patients is associated with a higher alcohol relapse rate, worse outcomes, including lower health-related quality of life and increased mortality [9, 10]. Crucially, psychiatric intervention has been shown to improve both liver-related and all-cause mortality among those with ALD and psychiatric comorbidities [11]. Recognizing these risks, the American Association for the Study of Liver Diseases (AASLD) emphasizes the importance of a multidisciplinary approach, including mental health evaluation, to ensure appropriate screening and treatment for patients with advanced ALD [12]. Identifying trends and high-risk subgroups with elevated or rising prevalence of psychiatric comorbidities is essential for optimizing clinical management and targeted intervention strategies.

Furthermore, liver disease itself is associated with psychiatric comorbidities, as suggested by data from the National Epidemiologic Survey on Alcohol and Related Conditions, showing individuals with liver disease from any cause have a higher risk of major depressive episode over the past 12 months compared to those without liver disease, even after adjusting for alcohol use [13]. In addition to ALD, other CLDs such as metabolic dysfunction-associated steatotic liver disease (MASLD) or chronic viral hepatitis are increasingly linked to psychiatric comorbidities [14]. Mendelian randomization studies support a causal relationship between MASLD and depression [15], and a Swedish cohort study found a significantly higher risk of severe depression in patients with MASLD (aHR: 1.8; 95% CI: 1.5–2.1) [16]. Moreover, a recent meta-analysis in 2024 estimated the prevalence of depression and anxiety in individuals with MASLD at 26.3% and 37.2%, respectively [17]. Unsurprisingly, hepatitis C has also been linked to high rates of depression and anxiety [18].

Despite the significance of psychiatric comorbidities in ALD, contemporary population-based studies characterizing national trends of psychiatric comorbidities among patients with ALD are lacking. Little is known about how these patterns differ by cirrhosis status or how ALD compares to other forms of CLD. We aim to address this gap by evaluating national trends over the past decade in the prevalence of nine key psychiatric comorbidities: substance use disorders, nicotine use disorder, personality disorders, major depressive disorder (MDD), anxiety disorder, post-traumatic stress disorder (PTSD), schizophrenia or psychotic disorders, bipolar disorder, and suicidal or homicidal ideation (SI/HI), among individuals with ALD. Additionally, comparative analyses were conducted between ALD and non-ALD CLD populations, both with and without cirrhosis. Subgroup analyses in the ALD cohort, stratified by cirrhosis status and age, further elucidate the trends in these clinically identifiable subgroups.

## Materials and Methods

### Study Population and Design

This population-level retrospective cohort study utilized de-identified electronic health records from the TriNetX database (TriNetX, LLC, Cambridge, MA), a real-world database integrating diagnostic records, procedures, laboratory data, and medication prescriptions. TriNetX includes over 113 million insured and uninsured individuals, covering both inpatient and outpatient records from 65 U.S. healthcare organizations (HCOs). These HCOs typically include large academic or research-focused medical centers providing inpatient, outpatient, and specialty care. The TriNetX platform has been validated for retrospective cohort studies and complies with HIPAA regulations [19, 20]. This study adhered to the Strengthening the Reporting of Observational Studies in Epidemiology (STROBE) reporting guideline for cohort studies.

We included patients aged ≥ 21 years diagnosed with ALD between January 1, 2015, and December 31, 2023. ALD was identified using ICD-10 codes (Supplementary material, sTable 1). Patients younger than 21 years were excluded. The index date was defined as the date of the first recorded ALD diagnosis. The primary outcome was the prevalence (%) of nine psychiatric disorders within one year of ALD diagnosis: substance use disorders, nicotine use disorder, personality disorders, MDD, anxiety disorder, PTSD, schizophrenia/psychotic disorders, bipolar disorder, and SI/HI, identified using ICD-10 codes.

### Definitions of Cohorts and Subgroup Analyses

Within the ALD cohort, two subgroup analyses were conducted based on cirrhosis status and age. Patients were stratified into three age groups: 21–39 years, 40–64 years, and ≥ 65 years. Non-cirrhotic ALD was defined as ALD or alcohol-associated hepatitis in the absence of cirrhosis or hepatic decompensation, including ascites, hepatic encephalopathy, or variceal bleeding.

To evaluate trends in psychiatric disorders among patients with ALD in comparison to individuals with non-ALD CLD, we established two control groups: (1) patients with non-ALD CLD without cirrhosis (e.g., chronic viral hepatitis) and [2] patients with non-ALD CLD with cirrhosis for comparative analyses. CLD was identified using ICD-10 codes, including viral hepatitis, autoimmune hepatitis, MASLD, MASH, and so on, and excluded any alcohol-associated liver disease (Supplementary material, sTable 1). We systematically analyzed and compared the prevalence of nine concurrent psychiatric disorders across different patient cohorts.

### Statistical Analyses

The age-standardized prevalence of ALD was estimated using the direct standardization method, with the 2020 U.S. Census population serving as the reference standard. Trends in the prevalence of psychiatric comorbidities over the study period were analyzed using piecewise log-linear regression models implemented in Joinpoint Regression Software (version 5.2.0.0) [21]. To assess temporal trends and identify significant shifts from 2015 to 2023, we calculated the annual percentage change (APC) and the average annual percentage change (AAPC), along with their corresponding 95% confidence intervals (CIs). Non-parallel AAPC comparisons between trends of subgroups were conducted using permutation tests and parametric methods. To ensure robust joinpoint estimation, a minimum of two observations was required from each joinpoint to the dataset’s endpoints, also with at least two observations between consecutive joinpoints [22].

## Results

Overall, the age-adjusted prevalence of confirmed diagnoses of ALD increased from 0.15% in 2015 to 0.21% in 2023. Among nine concurrent psychiatric disorders, MDD and anxiety were the most common in patients with ALD. In 2015, 28.54% of patients with ALD had comorbid MDD, rising to 36.37% in 2023. Similarly, anxiety was present in 28.12% of patients with ALD in 2015, increasing to 43.47% by 2023. Joinpoint analysis showed that anxiety, MDD, and PTSD showed significant upward trends, while the prevalence of schizophrenia significantly declined over the past decade.

### Trends and Comparisons in the Prevalence of Psychiatric Comorbidities in ALD, Stratified by Status of Cirrhosis

**Fig. 1:**
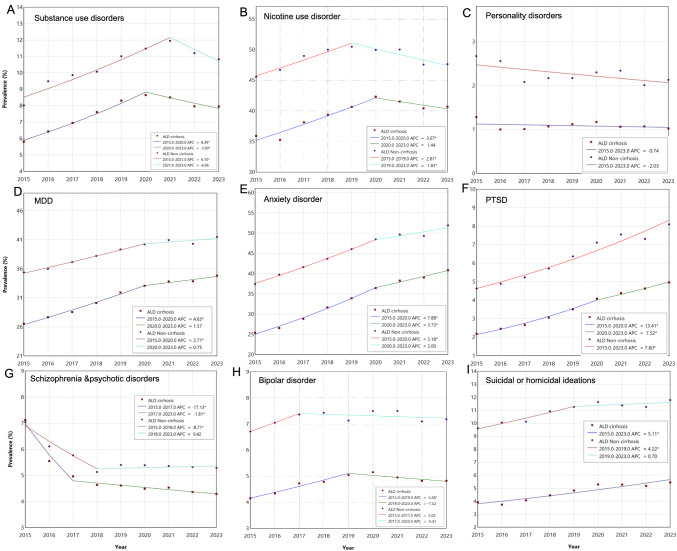
Trends of psychiatric disorders **A** substance use disorders, **B** nicotine use disorder, **C** personality disorders, **D** major depressive disorder (MDD), **E** anxiety disorder, **F** post-traumatic stress disorder (PTSD), **G** schizophrenia and psychotic disorders, **H** bipolar disorder, and **I** suicidal or homicidal ideations in patients with ALD with cirrhosis vs ALD without cirrhosis

Between 2015 and 2023, individuals with non-cirrhotic ALD consistently exhibited a higher prevalence of all nine psychiatric comorbidities compared to those with ALD cirrhosis, while those with cirrhosis showed a steeper increasing trend. For instance, in 2023, MDD and anxiety disorder were reported in 41.48% and 51.94% of 16,523 patients with non-cirrhotic ALD, respectively, compared to 34.82% and 40.85% of 55,186 patients with ALD cirrhosis Fig. 1A–I. Increasing prevalence trends were observed in those with ALD cirrhosis for 7 out of 9 psychiatric disorders examined: substance use disorders, nicotine use disorder, MDD, anxiety, PTSD, bipolar disorder, and SI/HI. Individuals with ALD with cirrhosis, compared to their counterparts without cirrhosis, had a higher rate of increasing prevalence in MDD, anxiety disorder, PTSD, and SI/HI. In contrast, the prevalence of schizophrenia and psychotic disorders decreased over the study period in both ALD subgroups, with a steeper rate of decrease in individuals with ALD cirrhosis (Table 1).

**Table 1:** Trends of concurrent psychiatric disorders in ALD patients stratified by cirrhosis status with APC, AAPC and non-parallel pair comparison

	Substance use disorders		Nicotine use disorder		Personality disorders		MDD		Anxiety disorder		PTSD		Schizophrenia and psychotic disorders		Bipolar disorder		Suicidal or homicidal ideations	
ALD patients with cirrhosis vs ALD patients without cirrhosis																		
	Cirrhosis	non-cirrhosis	Cirrhosis	non-cirrhosis	Cirrhosis	non-cirrhosis	Cirrhosis	non-cirrhosis	**Cirrhosis**	non-cirrhosis	Cirrhosis	non-cirrhosis	Cirrhosis	non-cirrhosis	Cirrhosis	non-cirrhosis	Cirrhosis	non-cirrhosis
Trend 1 Years APC, % (95%CI)	**2015–2020** 8.39* (7.05 to 9.76)	**2015–2021** 6.10* (3.27 to 9.01)	**2015–2020** 3.67* (1.74 to 5.63)	**2015–2019** 2.81* (1.16 to 4.48)	2015–2023 −0.74 (−2.80 to 1.37)	2015–2023 −2.03 (−4.15 to 0.14)	**2015–2020** 4.63* (3.72 to 5.56)	**2015–2020** 2.71* (1.82 to 3.61)	**2015–2020** 7.88* (6.59 to 9.19)	**2015–2020** 5.18* (4.04 to 6.34)	**2015–2020** 13.41* (11.73 to 15.12)	**2015–2023** 7.60* (6.25 to 8.97)	**2015–2017** −17.13* (−**20.62 to **−**13.48)**	**2015–2018** −8.71* (−**11.76 to **−**5.55)**	**2015–2019** 5.28* (2.97 to 7.63))	2015–2017 5.03 (−5.49 to 16.72)	**2015–2023** 5.11* (3.48 to 6.76)	2015–2019 4.22* (1.29 to 7.24)
Trend 2 Years APC, % (95%CI)	**2020–2023** −3.90* (−**6.55 to **−**1.18)**	2021–2023 −6.06 (−19.97 to 10.25	2020–2023 −1.44 (﻿−5.49 to 2.79)	**2019-** **2023** −1.83* (−**3.40 to **−**0.23)**	N/A	N/A	2020–2023 1.57 (−0.42 to 3.59)	2020–2023 0.75 (−1.19 to 2.73)	**2020–2023** 3.73* (0.96 to 6.57)	2020–2023 2.00 (−0.46 to 4.51)	**2020–2023** 7.52* (3.98 to 11.17)	N/A	**2017–2023** −1.81* (−**2.52 to **−**1.09)**	2018–2023 0.42 (−1.10 to 1.95)	2019–2023 −1.52 (−3.67 to 3.68))	2017–2023 −0.41 (−2.17 to 1.38)	N/A	2019–2023 0.70 (−2.14 to 3.61
AAPC, % 2015–2023 (95%CI)	3.61* **(2.66 to 4.57)**	2.92 (−0.29 to 6.23)	1.72* (0.32 to 3.14)	0.46 (−0.34 to 1.27)	−0.74 (−2.80 to 1.37)	−2.03 (−4.15 to 0.14)	3.47* (2.80 to 4.15)	1.97* (1.31 to 2.63)	6.30* (5.36 to 7.26)	3.98* (3.14 to 4.82)	11.16* (9.94 to 12.40)	7.60* (6.25 to 8.97)	−5.89* (−**6.69 to **−**5.08)**	−3.11* (−**4.19 to **−**2.02)**	1.82* (0.70 to 2.95)	0.92 (−1.16 to 3.05)	5.11* (3.48 to 6.76)	2.44* (0.99 to 3.91)
AAPC Comparison	0.69 (−2.71 to 4.90)		1.26 (−0.37 to 2.89)		1.29 (−1.19 to 3.77)		1.50* (0.56 to 2.44)		2.33* (1.06 to 3.59)		3.56* (1.89 to 5.22)		−2.78* (-4.13 to -1.43)		0.90 (−1.49 to 3.29)		2.66* (0.67 to 4.66)	

Bold font with *indicates statistical significance. *Significant at *P* < .05

*MDD* major depressive disorder, *PTSD* post-traumatic stress disorder, *ALD* alcohol-associated liver disease, *APC* annual percent change *AAPC* average annual percent change, *NA* not applicable *CI* confidence interval

#### Trends and Comparisons in the Prevalence of Psychiatric Comorbidities in ALD, Stratified by Age

**Fig. 2:**
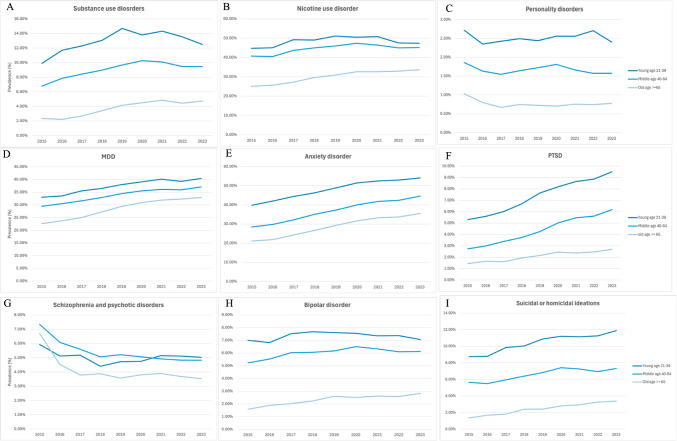
Trends of psychiatric disorders in patients with ALD, stratified by age group. **A** substance use disorders, **B** nicotine use disorder, **C** personality disorders, **D** major depressive disorder (MDD), **E** anxiety disorder, **F** post-traumatic stress disorder (PTSD), **G** schizophrenia and psychotic disorders, **H** bipolar disorder, and **I** suicidal or homicidal ideations. Age groups: young (21–39 years), middle-aged (40–64 years), and elderly (≥ 65 years)

Age-stratified analyses revealed that younger adults with ALD had the highest prevalence of concurrent psychiatric comorbidities Fig. 2A–I. In 2023, PTSD was diagnosed in 980 (9.50%) of young patients with ALD aged 21–39 years, compared to 2,716 (6.17%) of middle-aged patients aged 40–64 years, and 523 (2.70%) of older patients aged ≥ 65 years. A similar age-related pattern was observed for suicidal or homicidal ideation (SI/HI), with the highest prevalence among young adults (11.88%), followed by middle-aged (7.32%) and older adults (3.39%) in 2023. Notably, although older adults had the lowest absolute prevalence of psychiatric disorders across all disorders, they experienced substantial increases by AAPC over the study period in all psychiatric comorbidities except personality disorders, and schizophrenia and psychotic disorders.

In comparisons between the age groups, the trends for substance use disorders, nicotine use disorder, MDD, anxiety disorder, bipolar disorder, and SI/HI in individuals aged 65 years and older were significantly steeper compared to those observed in young patients. The differences in AAPC comparisons between younger and older ALD cohorts for these six psychiatric disorders were statistically significant (*p* < 0.05). Among older adults, the AAPC for substance use disorders was 10.43% (95% CI: 4.08% to 17.17%), nicotine use disorder was 3.87% (95% CI: 2.81% to 4.95%), MDD was 4.99% (95% CI: 4.11% to 5.89%), for anxiety was 7.00% (95% CI: 5.60% to 8.43%), for bipolar was 6.68% (95% CI: 4.52% to 8.88%), and for SI/HI was 12.37% (95% CI: 9.33% to 15.50%). In contrast, the AAPC among young ALD patients was 2.93% (95% CI: 0.24% to 5.69%) for substance use, 0.80% (95% CI: -0.74% to 2.37%) for nicotine use disorder, 2.57% (95% CI: 1.68% to 3.46%) for MDD, 3.86% (95% CI: 3.58% to 4.14%) for anxiety, 0.57% (95% CI: -1.14% to 2.32%) for bipolar, and 3.96% (95% CI: 2.85% to 5.08%) for SI/HI. However, older individuals had a marked decline in schizophrenia and psychotic disorders compared to their younger counterparts (Table 2).

**Table 2:** Trends of concurrent psychiatric disorders in ALD patients stratified by age with APC, AAPC and non-parallel pair comparison

	Substance use disorders			Nicotine use disorder			Personality disorders			MDD		
ALD patients, stratified by age (young, middle age, old)												
	Young	Middle	Old	Young	Middle	Old	Young	Middle	Old	Young	Middle	Old
Trend 1 Years APC, % (95%CI)	**2015–2019** 9.52* (3.86 to 15.94)	**2015–2020** 8.13* (5.54 to 10.78)	**2015–2020** 16.84*(7.83 to 26.60)	**2015–2019** 3.76* (0.60 to 7.02)	**2015–2020** 3.30* (1.59 to 5.04)	**2015-** **2020** 5.66*(4.20 to 7.14)	2015–2023 0.10 (−1.57 to 1.80)	2015–2023 −0.83 (−2.70 to 1.09)	**2015–2017** −17.35* (−**28.12 to **−**4.98)**	**2015–2020** 3.67* **(2.46 to 4.89)**	**2015–2020** 3.89* (3.15 to 4.63)	2015–2020 6.81* (5.59 to 8.05)
Trend 2 Years APC, % (95%CI)	2019–2023 −3.26 (−8.27 to 2.01)	2020–2023 −3.70 (−8.77 to 1.65)	2020–2023 0.52 (−15.99 to 20.28)	2019–2023 −2.07 (−5.05 to 1.01)	2020–2023 −1.97 (−5.57 to 1.76)	2020–2023 0.96 (−2.14 to 4.15)	N/A	N/A	2017–2023 1.87 (−0.51 to 4.30)	2020–2023 0.76 (−1.85 to 3.45)	2019–2023 1.18 (−0.42 to 2.80)	2020–2023 2.03 (−0.56 to 4.68)
AAPC, % 2015–2023 (95%CI)	2.93* (0.24 to 5.69)	3.53*(1.70 to 5.40)	10.43* (4.08 to 17.17)	0.80 (−0.74 to 2.37)	1.29*(0.05 to 2.55)	3.87* (2.81 to 4.95)	0.10 (−1.57 to 1.80)	-0.83 (−2.70 to 1.09)	-3.32* (−**5.96 to **−**0.61)**	2.57* (1.68 to 3.46)	2.87* (2.33 to 3.41)	4.99* (4.11 to 5.89)
AAPC Comparison (young vs middle)	−0.60 (3.90 to 2.70)			−0.49 (−2.49 to 1.51)			0.93 (−1.18 to 3 03)			−0.30 (−1.34 to 0.75)		
AAPC Comparison (young vs old)	−7.50* (−**14.59 to **−**0.41)**			−3.07* (−**4.95 to **−**1.18)**			3.42* (0.41 to 6.43)			−2.43* (−**3.68 to **−**1.17)**		

Anxiety disorder			PTSD			Schizophrenia and psychotic disorders			Bipolar disorder			Suicidal or homicidal ideations		
ALD patients, stratified by age (young, middle age, old)														
Young	Middle	Old	Young	Middle	Old	Young	Middle	Old	Young	Middle	Old	Young	Middle	Old
**2015–2020** 5.25* (4.86 to 5.63)	**2015–2020** 7.30* (6.10 to 8.52)	**2015–2020** 8.98* (7.05 to 10.95)	**2015–2021** 9.61*(7.02 to 12.25)	**2015–2021** 12.52* (10.79 to 14.27)	2015–2023 8.21* (6.22 to 10.24)	2015–2018 −7.83 (−15.16 to 0.14)	**2015–2017** −13.90* (−**19.28 to **−**8.16)**	**2015–2017** −23.58* (−**35.01 to **−**10.13)**	2015–2018 4.07 (−1.22 to 9.65)	**2015–2020** 4.02* (1.68 to 6.42)	**2015–2019** 11.54*(7.06 to 16.20)	**2015–2023** 3.96* (2.85 to 5.08)	2015–2020 6.01* **(2.67 to 9.45)**	**2015–2018** 19.30*(9.79 to 29.63)
**2020–2023** 1.58 (0.75 to 2.42) *	**2020–2023** 3.49*(0.90 to 6.14)	2020–2023 3.78 (−0.30 to 8.03)	2020–2023 4.98 (−0.47 to 10.74)	2021–2023 5.82 (−3.44 to 15.98)	N/A	2018–2023 2.62 (−1.11 to 6.50)	**2017–2023** −1.96* **(**−**3.03 to **−**0.89)**	2017–2023 −0.35 (−3.04 to 2.42)	2018–2023 −1.47 (−3.74 to 0.86)	2020–2023 −2.38 (−7.23 to 2.73)	2019–2023 2.03 (−2.07 to 6.30)	N/A	2020–2023 −0.27 (−7.17 to 7.14)	**2018–2023** 8.41*(4.46 to 12.52)
3.86* (3.58 to 4.14)	5.86*(4.98 to 6.74)	7.00* (5.60 to 8.43)	7.85* (5.97 to 9.77)	10.80* (8.81 to 12.83)	8.21* (6.22 to 10.24)	-1.43 (−4.09 to 1.31)	−5.09* (−**6.30 to **−**3.87)**	−6.75* (−**9.69 to **−**3.71)**	0.57 (−1.14 to 2.32)	1.58 (−0.12 to 3.30)	6.68* (4.52 to 8.88)	3.96* (2.85 to 5.08)	3.61*(1.19 to 6.09)	12.37* (9.33 to 15.50)
−2.00* (−**2.93 to **−**1.07)**			−2.95* (−**5.72 to **−**0.19)**			3.66* (0.71 to 6.62)			−1.00 (−3.44 to 1.43)			0.35 (−2.27 to 2.97)		
−3.15* (−**4.59 to **−**1.70)**			−0.36 (−2.89 to 2.16)			5.32* (1.29 to 9.35)			−6.10* (−**8.89 to **−**3.32)**			−8.41* (−**11.63 to **−**5.19)**		

Bold font with *indicates statistical significance. *Significant at *P* < .05

*MDD* major depressive disorder, *PTSD* post-traumatic stress disorder, *ALD* alcohol-associated liver disease, *APC* annual percent change *AAPC* average annual percent change, *NA* not applicable, *CI* confidence interval

Young age 21–39; Middle age 40–64; Old age >  = 65

### Trends and Comparisons in the Prevalence of Psychiatric Comorbidities in ALD Cirrhosis Versus Non-ALD CLD Cirrhosis

**Fig. 3:**
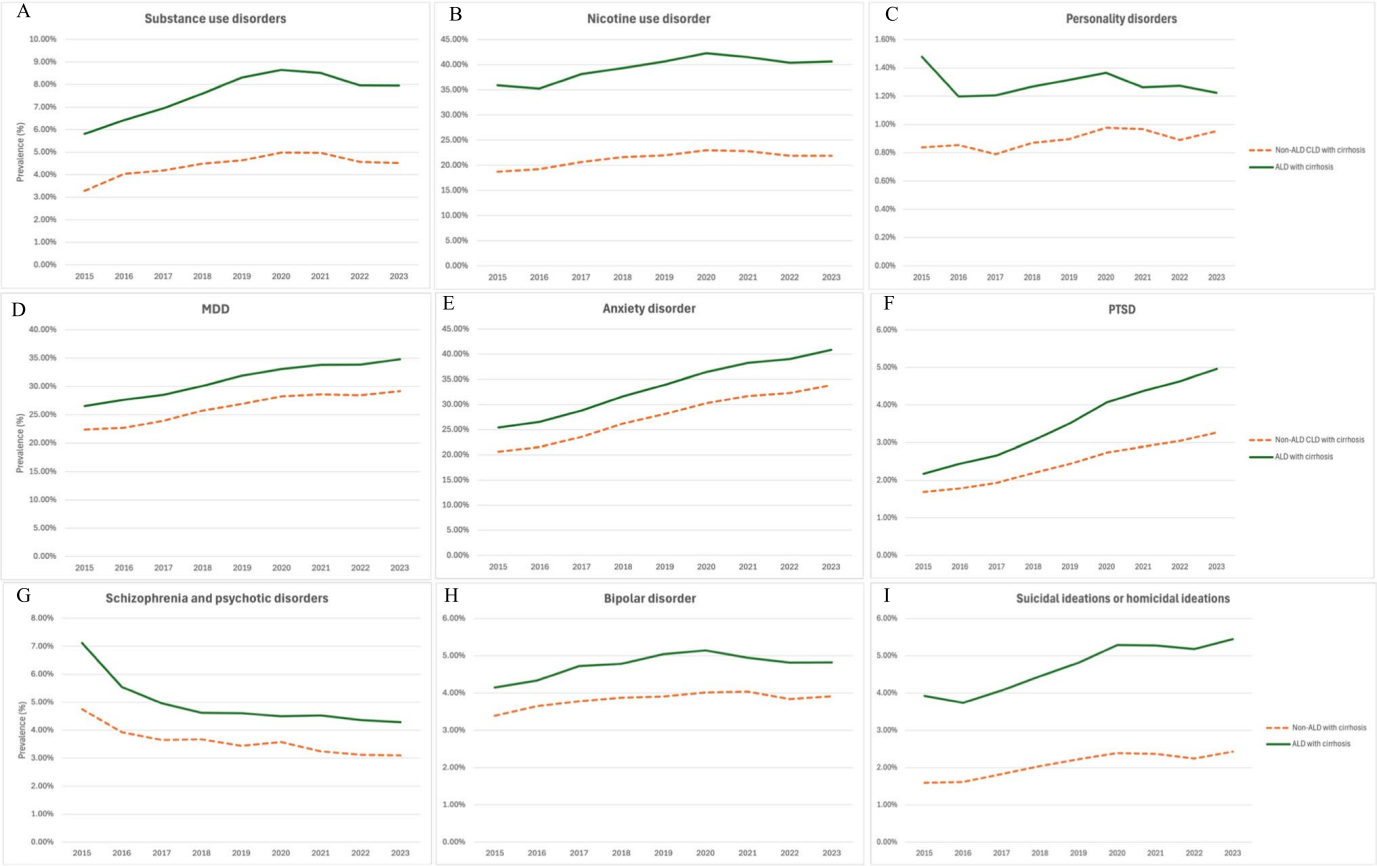
Trends of psychiatric disorders in patients with ALD cirrhotic vs non-ALD CLD cirrhotic, **A** substance use disorders, **B** nicotine use disorder, **C** personality disorders, **D** major depressive disorder (MDD), **E** anxiety disorder, **F** post-traumatic stress disorder (PTSD), **G** schizophrenia and psychotic disorders, **H** bipolar disorder, and **I** suicidal or homicidal ideations

Individuals with ALD cirrhosis exhibited a higher prevalence of nine concurrent psychiatric disorders than those with non-ALD CLD for the past decade Fig. 3A–I. Among individuals with ALD cirrhosis, anxiety disorder increased from 5,748 (25.40%) in 2015 to 22,487 (40.85%) in 2023; similarly, among non-ALD CLD cirrhosis patients, anxiety disorder rose from 12,262 (20.62%) to 46,131 (33.84%) over the same period. From 2015 to 2023, both cohorts exhibited significant upward trends in the prevalence of substance use disorders, nicotine use disorder, MDD, anxiety, PTSD, bipolar disorder, and SI/HI. The AAPCs for ALD cirrhosis were 3.61% for substance use disorders, 1.72% for nicotine use disorder, 3.47% for MDD, 6.30% for anxiety disorder, 11.16% for PTSD, 1.82% for bipolar, and 5.11% for SI/HI, while the corresponding AAPCs for non-ALD CLD cirrhosis were 3.05%, 1.89%, 3.53%, 6.55%, 9.21%, 1.70%, and 5.36% respectively (Table 3).

**Table 3:** Trends of concurrent psychiatric disorders in ALD patients with cirrhosis vs non-ALD CLD with cirrhosis with APC, AAPC and non-parallel pair comparison

	Substance use disorders		Nicotine use disorder		Personality disorders		MDD		Anxiety disorder	
ALD patients with cirrhosis vs non-ALD CLD patients with cirrhosis										
	ALD	CLD	ALD	CLD	ALD	CLD	ALD	CLD	ALD	CLD
Trend 1 Years APC, % (95%CI)	**2015–2020** 8.39* (7.05 to 9.76)	2015–2020 7.69* (2.84 to 12.77)	**2015–2020** 3.67*(1.74 to 5.64)	**2015–2020** 4.32* (2.86 to 5.80)	2015–2023 −0.74 (−2.80 to 1.37)	2015–2023 1.95* (0.35 to 3.58)	**2015–2020** 4.63* (3.72 to 5.56)	2015–2020 5.11* (3.58 to 6.67)	**2015–2020** 7.88* (6.59 to 9.19)	**2015–2020** 8.43* (6.90 to 9.98(
Trend 2 Years APC, % (95%CI)	**2020–2023** −3.90* (−**6.55 to -1.18)**	2020–2023 −4.24 (−13.62 to 6.16)	2020–2023 −1.44 (−5.49 to 2.79)	2020–2023 −2.05 (−5.06 to 1.08)	N/A	N/A	2020–2023 1.57 (−0.42 to 3.59)	2020–2023 0.96 (−2.31 to 4.34)	**2020–2023** 3.73* (0.96 to 6.57)	**2020–2023** 3.51* (0.28 to 6.85)
AAPC, % 2015–2023 (95%CI)	3.61* (2.66 to 4.57)	**3.05 (**−**0.40 to 6.62)**	1.72* (0.32 to 3.14)	1.89* (0.83 to 2.95)	−0.74 (−2.80 to 1.37)	1.95* (0.35 to 3.58)	3.47* (2.80 to 4.15)	3.53* (2.42 to 4.66)	6.30* (5.36 to 7.26)	6.55* (5.44 to 7.68)
AAPC Comparison	0.56 (−3.08 to 4.19)		−0.16 (−1.93 to 1.60)		−**2.69 (**−**4.88 to **−**0.51)**		−0.06 (−1.37 to 1.25)		−0.25 (−1.71 to 1.21)	

PTSD		Schizophrenia and psychotic disorders		Bipolar disorder		Suicidal or homicidal ideations	
ALD patients with cirrhosis vs non-ALD CLD patients with cirrhosis							
ALD	CLD	ALD	CLD	ALD	CLD	ALD	CLD
**2015–2020** 13.41* (11.73 to 15.12)	**2015–2023** 9.21* (8.16 to10.27)	**2015–2017** −17.13* (−**20.62 to **−**13.48)**	2015–2017 −10.84 (−20.86 to 0.46)	**2015–2019** 5.28* (2.97 to 7.63))	**2015–2018** 4.77* (0.52 to 9.20)	**2015–2023** 5.11* (3.48 to 6.76)	**2015–2020** 9.03*(5.24 to 12.94)
**2020–2023** 7.52* (3.98 to 11.17)	N/A	**2017–2023** −1.81* (−**2.52 to **−**1.09)**	**2017–2023** −2.81* (−**4.74 to **−**0.83)**	2019–2023 −1.52 (−3.67 to 3.68))	2018–2023 −0.10 (−1.93 to 1.77)	N/A	2020–2023 −0.47 (−8.02 to 7.71)
11.16* (9.94 to 12.40)	9.21* (8.16 to10.27)	−5.89* (−**6.69 to **−**5.08)**	−**4.88 (**−**7.10 to **−**2.61)**	1.82* (0.70 to 2.95)	1.70* (0.32 to 3.10)	5.11* (3.48 to 6.76)	5.36* (2.65 to 8.15)
1.95* (0.45 to 3.46)		−1.01 (−3.39 to 1.37)		0.12 (−1.67 to 1.91)		−0.26 (−3.32 to 2.80)	

Bold font with *indicates statistical significance. *Significant at P < .05

*MDD* major depressive disorder, *PTSD* post-traumatic stress disorder, *ALD* alcohol-associated liver disease, *CLD* chronic liver disease, *APC* annual percent change, *AAPC* average annual percent change, *NA* not applicable, *CI* confidence interval

We performed the same analysis comparing the prevalence and trends of psychiatric comorbidities among those without cirrhosis with ALD versus CLD and found that the prevalence of psychiatric comorbidities among those with ALD are higher than those with non-ALD CLD (Supplementary material, sFigure 1). In this population without cirrhosis, both ALD and CLD had an upward trend in comorbid MDD, anxiety, PTSD, and SI/HI, and a lower trend of schizophrenia and psychotic disorders by AAPC. Notably, the rate of increase of anxiety disorder among individuals with non-ALD CLD was higher than those with ALD by 1.72% (Supplementary material, sTable2).

## Discussion

This is the first large-scale, population-based study to comprehensively examine the prevalence and temporal trends of nine concurrent psychiatric comorbidities among patients with ALD and non-ALD CLD, stratified by cirrhosis status. Our findings indicate that individuals with ALD without cirrhosis exhibited a higher prevalence of all nine psychiatric disorders compared to those with ALD with cirrhosis and non-ALD CLD. Furthermore, significant increases in the prevalence of MDD, anxiety disorder, and PTSD were observed over the past decade in both the ALD and non-ALD CLD groups, regardless of cirrhosis status. When stratified by age, we found older adults with ALD had an increasing prevalence of all psychiatric disorders except personality disorders and schizophrenia and psychotic disorders during the study period, although overall prevalence remained lower than in other age groups.

In our study, the age-standardized prevalence of confirmed ALD diagnoses was lower than that reported in prior NHANES-based studies [23, 24]. This difference may be explained by the broad NHANES study definition of ALD as those meeting steatotic liver disease by vibration-controlled transient elastography and a self-reported alcohol intake. This definition is likely to overestimate disease prevalence of ALD because only a minority of those meeting the alcohol threshold that defines ALD in the updated AASLD Delphi consensus [alcohol consumption > 350 g/week (women)/ > 420 g/week (men)] develop clinically significant ALD, such as those captured in our cohort [25].

We found that individuals with ALD and cirrhosis exhibited a lower prevalence of concurrent psychiatric comorbidities compared to those without cirrhosis. This observation may be explained by cirrhosis frequently being diagnosed during decompensation or hospitalization, when longstanding psychiatric conditions may remain unrecognized due to limited healthcare access and low health literacy in this group of patients. Physicians might also prioritize severe medical issues, such as esophageal varices or hepatic encephalopathy in decompensated cirrhosis, and these patients may also present with more critical medical conditions that mask anxiety or depression. Nevertheless, the increasing prevalence of multiple psychiatric disorders, including substance use disorders, MDD, anxiety disorder, and PTSD in ALD, regardless of cirrhosis over the study period, suggests the burden of disease is likely to rise in the future.

Consistent with previous literature indicating that alcohol use disorders, anxiety, and PTSD frequently co-occur in young adults, we also found that younger patients with ALD had the highest prevalence of psychiatric comorbidities, followed by middle-aged individuals, with the lowest prevalence observed in older adults [26, 27]. Chen et al. showed that younger patients experienced more severe depression before liver transplantation and poorer family functioning than older patients in a retrospective study from Taiwan [27]. This finding aligns with prior research by Jinjuvadia et al. [28], who identified younger age as an independent predictor of psychiatric disorders, including depression and anxiety, among hospitalized patients with ALD. Although older ALD patients in our analysis demonstrated a lower overall prevalence of psychiatric comorbidities, the rates of increase over the past decade for conditions including substance use disorders, nicotine use disorders, MDD, anxiety, bipolar disorder, and SI/HI were significantly steeper than those observed in younger individuals. These age-related trends have not been addressed in prior studies and highlight the need for increased vigilance in the care of older adults with ALD, who are projected to have a higher burden of psychiatric comorbidities, especially with an aging population.

In our study, both MDD and anxiety showed significant increases over the past 10 years in the non-ALD CLD group. However, when evaluating the prevalence of concurrent psychiatric comorbidities, the non-ALD CLD group continued to exhibit lower rates across all nine disorders compared to patients with ALD over the past decade. Although the association between depression and chronic liver disease is increasingly acknowledged, few studies have directly examined long-term trends in psychiatric comorbidities, such as anxiety, PTSD, bipolar disorder, or SI/HI between patients with ALD and those with non-ALD CLD. A prior retrospective study using data from the National Inpatient Sample reported significantly higher rates of anxiety disorder, PTSD, and depression among hospitalized patients with ALD than those with chronic liver diseases not caused by alcohol (all *p* < 0.05) [28]. This is consistent with our findings in terms of the absolute prevalence of psychiatric comorbidities being higher in ALD than in non-ALD CLD. However, individuals with non-ALD CLD without cirrhosis in our study had an even higher rate of increase in anxiety disorder compared to their counterparts with ALD over the study period. This finding may represent anxiety disorders among individuals with MASLD, since there has been a significant rise in the prevalence of noncirrhotic MASLD over the past 20 years.

However, there remains a lack of specific data on PTSD and other psychiatric comorbidities in MASLD and HCV populations, highlighting an important gap in the literature. Our study contributes an additional insight in evaluating and comparing the prevalence and temporal trends of nine concurrent psychiatric disorders in ALD vs non-ALD CLD stratified by cirrhosis status.

In terms of trends, our analysis revealed significant increases in MDD, anxiety disorder, PTSD, and substance use disorders in patients with ALD over the past decade, particularly between 2015 and 2019. Similarly, Patel et al. presented the rising prevalence of generalized anxiety disorder in liver cirrhosis hospitalizations, which increased from 0.17% in 2009 to 0.92% in 2019, and that of depression increased from 7 to 12% during the same period [29]. However, that study did not apply Joinpoint regression modeling to assess the statistical significance of these temporal shifts. Our findings are consistent with the findings from Patel et al. and suggest the rates of anxiety disorder have continued to rise since 2019, albeit at a slower pace than from 2015 to 2020. Additionally, our study revealed that the decreasing trends in schizophrenia and psychotic disorders may be related to increased post-discharge psychiatric referrals [30], growing recognition of the gut–liver–brain axis [31], and the implementation of integrated care models, which collectively enhance diagnostic accuracy and enable more tailored psychiatric management in ALD populations.

This study has several strengths. First, it provides an updated, population-level analysis incorporating data from over 113 million individuals across the United States, with both inpatient and outpatient records. Unlike prior research that primarily focused on depression, anxiety, PTSD, or suicidal ideation, our investigation comprehensively included the nine most prevalent psychiatric comorbidities among patients with ALD over the past decade. Second, our inclusion of uninsured individuals adds valuable insight, as most large databases do not include this population. Additionally, the use of Joinpoint analysis enables the evaluation of the temporal trend of psychiatric comorbidities in this vulnerable population. Despite these strengths, our study has limitations. Although the use of ICD-10 codes is a validated approach for identifying ALD and cirrhosis [32], the possibility of misclassification and missing data, which are common limitations of retrospective administrative database studies. Additionally, MetALD was not discussed in our study, as our study period (2015–2023) largely predates the formal introduction of this definition. Detailed geographic and insurance-related data are limited in the TriNetX database and therefore could not be stratified individually. Moreover, the COVID-19 pandemic may have influenced both the access to psychiatric services and alcohol consumption behaviors, potentially contributing to the observed trend shifts between 2020 and 2022. Future studies with socioeconomic data are needed to evaluate the association of psychiatric comorbidities from these trends and clinical outcomes. Lastly, socioeconomic factors and alcohol consumption levels were not accounted for in our analysis due to limitations of the dataset.

## Conclusion

Our study highlights a high prevalence and a significant upward trend in MDD, anxiety, and PTSD among patients with ALD over the past decade, with the most pronounced increases observed in older individuals and those with cirrhosis. These findings provide a contemporary, nationwide perspective, underlining the importance for clinicians to proactively address psychiatric comorbidities in ALD patients to improve both physical and mental health outcomes.

## Supplementary Information

Below is the link to the electronic supplementary material.

Supplementary file1 (DOCX 1306 KB)

## Data Availability

The aggregated data from TriNetX that support the findings of this study are available from the corresponding author, Dr. Peng-Sheng (Brian) Ting, upon reasonable request.
