# Supplementary material for: Trends in Concurrent Psychiatric Comorbidities in Alcohol-Associated Liver Disease: A Nationwide Study from 2015–2023
Source: Dig Dis Sci. 2025 Nov 6;71(4):1493–504. doi: 10.1007/s10620-025-09494-x (PMC13144217; doi:10.1007/s10620-025-09494-x)
Supplement: Supplementary file 1 — Supplementary file1 (DOCX 1306 KB) [file 10620_2025_9494_MOESM1_ESM.docx]

Supplementary Materials

**sTable1. ICD10-CM diagnosis codes for each cohort**

| Disease | ICD-9/10-CM diagnosis codes/procedure codes/ RxNorm codes |
| --- | --- |
| ALD | K70 (Alcoholic liver disease), K70.1 (Alcoholic hepatitis), K70.10 (Alcoholic hepatitis without ascites), K70.11 Alcoholic hepatitis with ascites), **K70.2 (Alcoholic fibrosis and sclerosis of liver),** K70.3 (Alcoholic cirrhosis of liver), K70.30 (Alcoholic cirrhosis of liver without ascites), K70.31 (Alcoholic cirrhosis of liver with ascites), K 70.4 (Alcoholic hepatic failure), K70.9 (Alcoholic liver disease, unspecified) |
| ALD with cirrhosis | K70.3 (Alcoholic cirrhosis of liver), K70.30 (Alcoholic cirrhosis of liver without ascites), K70.31 (Alcoholic cirrhosis of liver with ascites) |
| ALD without cirrhosis | K70 (Alcoholic liver disease), K70.1 (Alcoholic hepatitis), K70.10 (Alcoholic hepatitis without ascites), **K70.2 (Alcoholic fibrosis and sclerosis of liver),** K70.4 (Alcoholic hepatic failure), or K70.9 (Alcoholic liver disease, unspecified)  and **exclude** K70.3 (Alcoholic cirrhosis of liver), R18 (ascites), K76.82 (hepatic encephalopathy), or I85 (Esophageal varices) |
| CLD without cirrhosis | B18 (Chronic hepatitis), B18.0 (Chronic viral hepatitis B with delta-agent), B18.1 (Chronic viral hepatitis B without delta-agent Chronic (viral) hepatitis B) , B18.2 (Chronic viral hepatitis C), B18.8 (Other chronic viral hepatitis), B18.9 (Chronic viral hepatitis, unspecified), B19 (unspecified chronic viral hepatitis), B19.0 (Unspecified viral hepatitis with hepatic coma), B19.9 (Unspecified viral hepatitis without hepatic coma), E83.01 (Wilson’s disease), E83.110 (Hereditary hemochromatosis), K73 (chronic hepatitis, not elsewhere classified), K73.0 (Chronic persistent hepatitis, not elsewhere classified), K73.1 (Chronic lobular hepatitis, not elsewhere classified), K73.2 (Chronic active hepatitis, not elsewhere classified), K73.8 (Other chronic hepatitis, not elsewhere classified), K73.9 (Chronic hepatitis, unspecified), K74.0 (Hepatic fibrosis), K74.1 (Hepatic sclerosis), K74.2 (Hepatic fibrosis with hepatic sclerosis), K75 (other inflammatory disease of liver), K75.3 (Granulomatous hepatitis), K75.4 (autoimmune hepatitis), K75.8 (Other specified inflammatory liver diseases), K75.81 (NASH), K75.9 (Inflammatory liver disease, unspecified), K76 (other disease of liver) K76.0 (Fatty (change of) liver, not elsewhere classified), K76.1 (Chronic passive congestion of liver), K76.9 (Liver disease, unspecified), or K83.01 (Primary sclerosing cholangitis)and **exclude** K70 (Alcoholic liver disease), K70.1 (Alcoholic hepatitis), K70.10 (Alcoholic hepatitis without ascites), K70.11 Alcoholic hepatitis with ascites), **K70.2 (Alcoholic fibrosis and sclerosis of liver),** K70.3 (Alcoholic cirrhosis of liver), K70.30 (Alcoholic cirrhosis of liver without ascites), K70.31 (Alcoholic cirrhosis of liver with ascites), K 70.4 (Alcoholic hepatic failure), or K70.9 (Alcoholic liver disease, unspecified) |
| CLD with cirrhosis | Anything above in CLD and K74 (fibrosis and cirrhosis of liver), K74.3 (Primary biliary cirrhosis), K74.4 (Secondary biliary cirrhosis), K74.5(Biliary cirrhosis, unspecified), K74.6 (Other and unspecified cirrhosis of liver), K74.60 (unspecified cirrhosis of liver), or K74.69 (other cirrhosis over liver)  and **exclude** K70 (Alcoholic liver disease), K70.1 (Alcoholic hepatitis), K70.10 (Alcoholic hepatitis without ascites), K70.11 Alcoholic hepatitis with ascites), **K70.2 (Alcoholic fibrosis and sclerosis of liver),** K70.3 (Alcoholic cirrhosis of liver), K70.30 (Alcoholic cirrhosis of liver without ascites), K70.31 (Alcoholic cirrhosis of liver with ascites), K 70.4 (Alcoholic hepatic failure), K70.9 (Alcoholic liver disease, unspecified) |


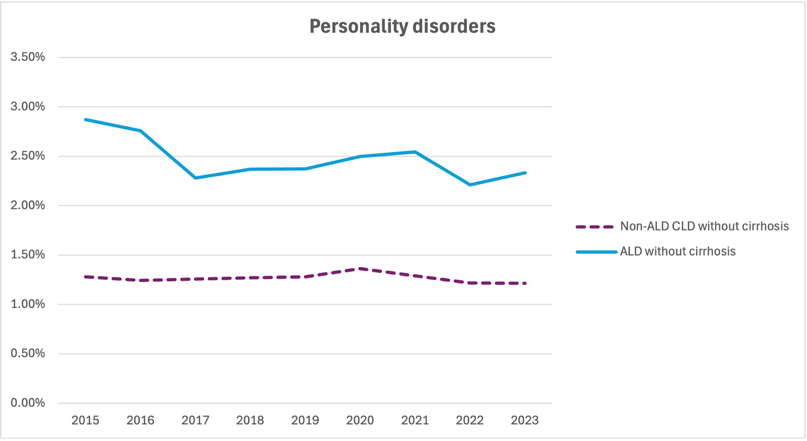

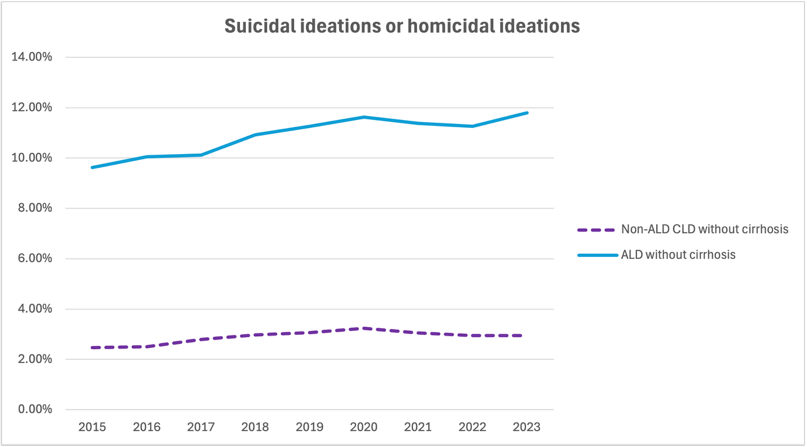

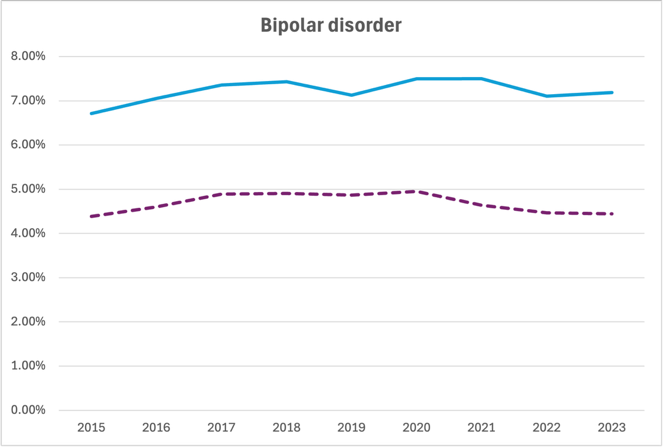

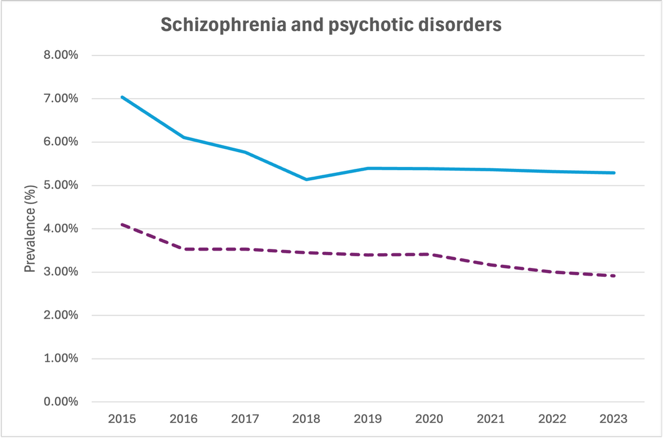

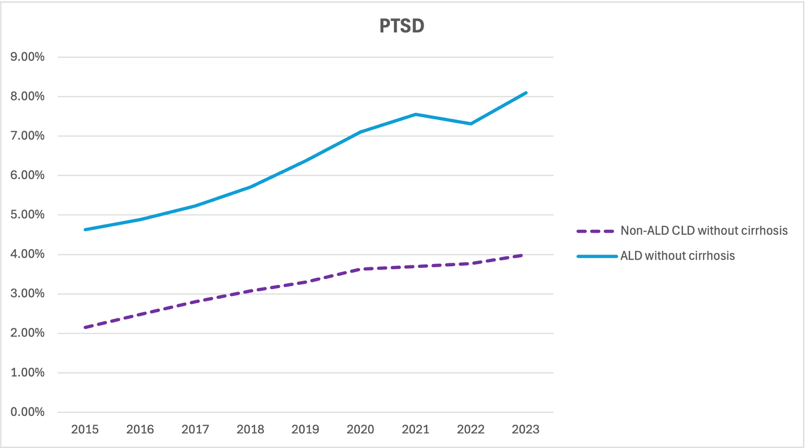

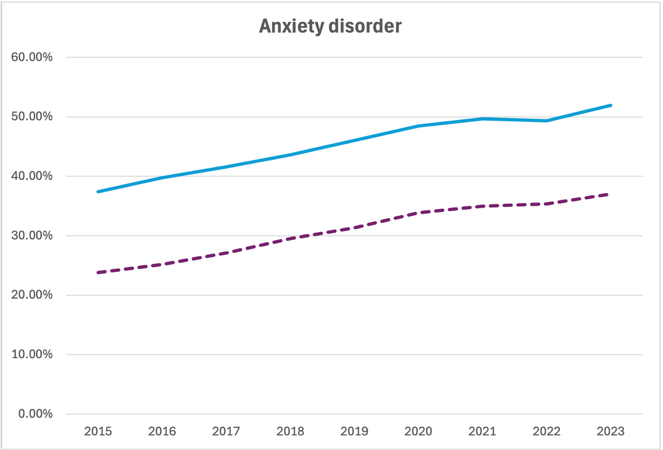

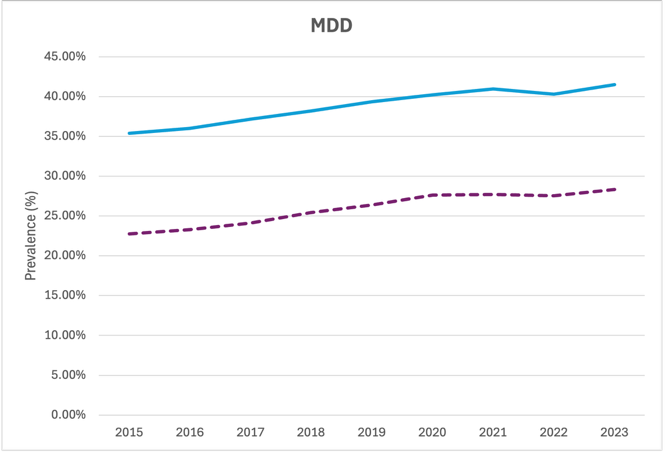

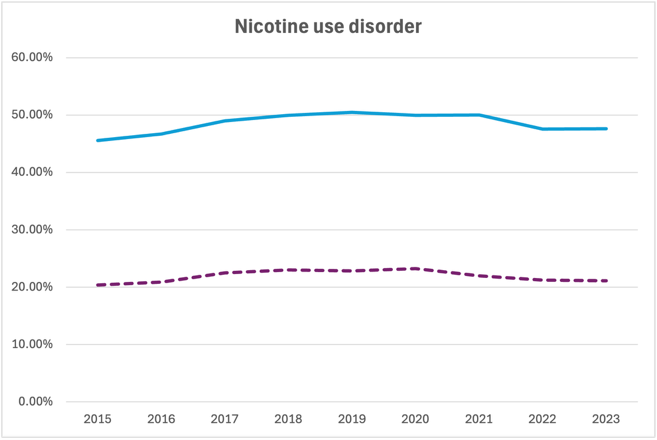

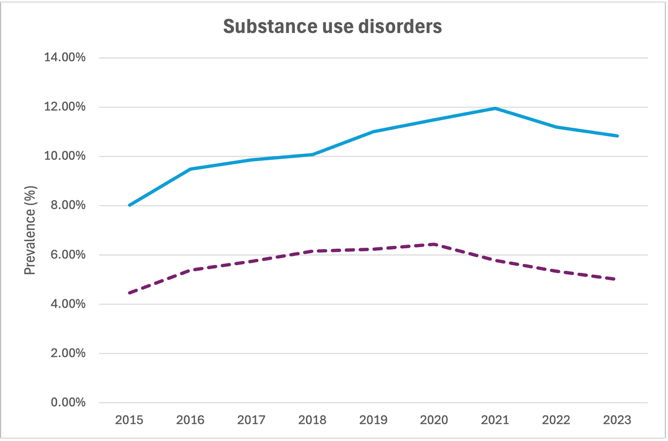


A

B

C

D

E

F

G

H

I

**sFigure 1.** Trends of psychiatric disorders in patients with ALD noncirrhotic vs non-ALD CLD noncirrhotic, (A) Substance use disorders, (B) Nicotine use disorder, (C) Personality disorders, (D) Major depressive disorder (MDD), (E) Anxiety disorder, (F) Post-traumatic stress disorder (PTSD), (G) Schizophrenia and psychotic disorders, (H) Bipolar disorder, and (I) Suicidal or homicidal ideations.

**sTable 2. Trends of concurrent psychiatric disorders in ALD patients without cirrhosis vs non-ALD CLD without cirrhosis with APC, AAPC and non-parallel pair comparison**

|  | Substance use disorders | | Nicotine use disorder | | Personality disorders | | MDD | | Anxiety disorder | | PTSD | | Schizophrenia and psychotic disorders | | Bipolar disorder | | Suicidal or homicidal ideations | |
| --- | --- | --- | --- | --- | --- | --- | --- | --- | --- | --- | --- | --- | --- | --- | --- | --- | --- | --- |
| **ALD patients without cirrhosis vs non-ALD CLD patients without cirrhosis** | | | | | | | | | | | | | | | | | | |
|  | ALD | CLD | ALD | CLD | ALD | CLD | ALD | CLD | ALD | CLD | ALD | CLD | ALD | CLD | ALD | CLD | ALD | CLD |
| Trend 1  Years  APC, %  (95%CI) | **2015-2021**  **6.10* (3.27 to 9.01)** | **2015-2019**  **8.94* (4.43 to 13.63)** | **2015-2019**  **2.81* (1.16 to 4.48)** | **2015-2018**  **4.97* (0.89 to 9.21)** | 2015-2023  -2.03 (-4.15 to 0.14) | 2015-2023  -0.21 (-1.27 to 0.87) | **2015-2020**  **2.71* (1.82 to 3.62)** | **2015-2020**  **4.02* (3.04 to 5.01)** | **2015-2020**  **5.18* (4.04 to 6.34)** | **2015-2020 7.43* (6.45 to 8.43)** | **2015-2023**  **7.60* (6.25 to 8.97)** | **2015-2018 13.45* (8.78 to 18.31)** | **2015-2018**  **-8.71* (-11.76 to -5.55)** | **2015-2023**  **-3.42* (-4.51 to-2.31)** | 2015-2017  5.03 (-5.49 to 16.72) | **2015-2018**  **4.41* (0.43 to 8.55)** | **2015-2019 4.22* (1.29 to 7.24)** | **2015-2020 5.65*(3.58 to 7.76)** |
| Trend 2  Years  APC, %  (95%CI) | 2021-2023  -6.06 (-19.97 to 10.25) | **2019-2023**  **-6.69* (-10.54 to 2.66)** | **2019-2023**  **-1.83* (-3.40 to -0.23)** | **2018-2023 -2.01*(-3.73 to -0.26)** | N/A | N/A | 2020-2023  0.75 (-1.19 to 2.73) | 2020-2023 0.83 (-1.29 to 2.99) | **2020-2023**  2.00 (-0.46 to 4.51) | **2020-2023 2.87* (0.77 to 5.02)** | N/A | **2018-2023 4.96* (3.01 to 6.96)** | 2018-2023  0.42 (-1.10 to 1.95) | N/A | 2017-2023  -0.41 (-2.17 to 1.38) | **2018-2023**  **-2.47* (-4.15 to -0.76)** | 2019-2023  0.70 (-2.14 to 3.61 | 2020-2023  -3.65 (-7.82 to 0.72) |
| AAPC, %  2015-2023  (95%CI) | 2.92 (-0.29 to 6.23) | 0.82 (- 1.28 to 2.97) | 0.46 (-0.34 to 1.27) | 0.55 (-0.76 to 1.87) | -2.03 (-4.15 to 0.14) | -0.21 (-1.27 to 0.87) | **1.97* (1.32 to 2.63)** | **2.81* (2.09 to 3.53)** | **3.98* (3.14 to 4.82)** | **5.70* (4.98 to 6.42)** | **7.60* (6.25 to 8.97)** | **8.07* (6.58 to 9.58)** | **-3.11 (-4.14 to -2.02)** | **-3.42* (-4.51 to-2.31)** | 0.92 (-1.16 to 3.05) | 0.06 (-1.22 to 1.35) | **2.44* (0.99 to 3.91)** | **2.07*(0.58 to 3.57)** |
| AAPC  Comparison | 2.10 (-1.80 to 5.99) | | -0.09 (-1.63 to 1.46) | | -1.82 (-3.81 to 0.16) | | -0.84 (-1.81 to 0.13) | | **-1.72* (-2.83 to -0.62)** | | -0.47 (-2.34 to 1.41) | | 0.31 (-1.11 to 1.73) | | 0.87 (-1.60 to 3.33) | | 0.38 (-1.71 to 2.47) | |

Bold font with *indicates statistical significance. *Significant at P < .05.

MDD: major depressive disorder; PTSD: post-traumatic stress disorder; ALD: alcoholic liver disease; CLD: chronic liver disease; APC: annual percent change; AAPC: average annual percent change; NA: not applicable; CI: confidence interval
